# Supervised non-negative matrix factorization methods for MALDI imaging applications

**DOI:** 10.1093/bioinformatics/bty909

**Published:** 2018-11-05

**Authors:** Johannes Leuschner, Maximilian Schmidt, Pascal Fernsel, Delf Lachmund, Tobias Boskamp, Peter Maass

**Affiliations:** Department of Mathematics, Center for Industrial Mathematics, University of Bremen, Bremen, Germany

## Abstract

**Motivation:**

Non-negative matrix factorization (NMF) is a common tool for obtaining low-rank approximations of non-negative data matrices and has been widely used in machine learning, e.g. for supporting feature extraction in high-dimensional classification tasks. In its classical form, NMF is an unsupervised method, i.e. the class labels of the training data are not used when computing the NMF. However, incorporating the classification labels into the NMF algorithms allows to specifically guide them toward the extraction of data patterns relevant for discriminating the respective classes. This approach is particularly suited for the analysis of mass spectrometry imaging (MSI) data in clinical applications, such as tumor typing and classification, which are among the most challenging tasks in pathology. Thus, we investigate algorithms for extracting tumor-specific spectral patterns from MSI data by NMF methods.

**Results:**

In this article, we incorporate a priori class labels into the NMF cost functional by adding appropriate supervised penalty terms. Numerical experiments on a MALDI imaging dataset confirm that the novel supervised NMF methods lead to significantly better classification accuracy and stability as compared with other standard approaches.

**Availability and implementaton:**

https://gitlab.informatik.uni-bremen.de/digipath/Supervised_NMF_Methods_for_MALDI.git

**Supplementary information:**

Supplementary data are available at *Bioinformatics* online.

## 1 Introduction

The pathological diagnosis of a tumor found in a tissue specimen, including the determination of the tumor origin and genetic subtype, is crucial for individualized therapy decision and accurate prognosis. This task, often referred to as tumor typing, is conventionally based on microscopic examination of stained tissue sections, as well as molecular or genetic tests (Klauschen *et al*., 2015; Kruglyak *et al*., 2014), and often is subtle and requires extensive training and experience.

Naturally, since the early stages of digital pathology, large efforts have been devoted to support expert diagnosis by statistical and computational methods (Pantanowitz *et al*., 2010). Moreover, mass spectrometry imaging (MSI), which has been established over the last decade as a routine methodology in analytical chemistry and proteomics research, has demonstrated a high potential for a variety of tasks in clinical pathology (Kriegsmann *et al*., 2015; Schwamborn and Caprioli, 2010). However, the high complexity and large data volumes of MSI experiments demand for appropriate, dedicated computational analysis tools.

Recent reports on applications of MSI to tumor typing are the starting point for our present research (Casadonte *et al*., 2014; Kriegsmann *et al*., 2015; Veselkov *et al*., 2014). The proposed methods primarily rely on the extraction of spectral features (single peaks or more complex spectral patterns) from large sets of training data. In the mentioned sources, both statistical tests for detecting discriminative spectral features, as well as computational methods, such as principal component analysis (PCA), probabilistic latent semantic analysis (PLSA) or non-negative matrix factorization (NMF) have been applied. These spectral features then form the basis for constructing a subsequent classification scheme (LDA, logistic regression, etc.). In particular, spectral patterns based on NMF decomposition have been demonstrated to result in competitive or even improved classification schemes for different tumor typing tasks (Boskamp *et al*., 2016).

These results on NMF-based tumor typing rely on a first unsupervised step for extracting spectral patterns followed by a subsequent supervised training of a classification model. In this article, however, we aim at (i) motivating and analyzing classification schemes based on supervised NMF, and (ii) evaluating the potential of such methods for tumor typing using matrix-assisted laser desorption/ionization (MALDI) MSI.

Concerning the first aim, we will combine the NMF data decomposition with the construction of the classification scheme in a unified approach. We will do this in the context of regularized NMF decompositions, where the classification error for either logistic regression or linear discriminant analysis (LDA) is added as a separate penalty term.

Concerning the second aim, we will evaluate the derived classification schemes using MALDI MSI data from a collection of tissue microarrays (TMAs) of different types of lung cancer tissue samples. Due to the comparatively large number of patients (*N* = 304), this challenging dataset exhibits a large biological variation, as well as apparent technical variation between the different measurements. According to our main hypothesis, we expect that the spectral patterns determined by the supervised NMF methods are more relevant for the respective classification task, as compared with the unsupervised NMF approach, and hence result in a higher classification accuracy.

The article is organized as follows: Section 2 describes a general and well-established approach to regularized NMF functionals and their use in classification contexts. In Section 3, we introduce our proposed supervised NMF models and corresponding algorithms. In Section 4, we present the results of extensive numerical experiments evaluating the different variants of supervised NMF and classification schemes. The final section is devoted to a discussion of the results and an outlook on future research directions.

## 2 Non-negative matrix factorization

Matrix factorization methods address the task of computing a low-rank approximation to a given, typically large data matrix *Y* of dimension n×m. In matrix notation, this requires to determine matrices *K* and *X* of dimensions *n* × *p* and *p* × *m*, respectively, such that p≪m,n and Y≈KX. Such methods are the basis for a large variety of applications including compression, feature extraction or basis learning (Golub and Van Loan, 1996; Lee and Seung, 2001). If the data are non-negative, as it is the case for MALDI MSI, it is often desirable to request that *K* and *X* are also non-negative, leading to a NMF.

In a standard situation, the data matrix *Y* combines *n* data vectors of length *m* (rows of data matrix *Y*), which are related to *n* entities under consideration (e.g. MALDI spectra). Computing an NMF helps to determine *p* characteristic non-negative basis vectors that allow to approximate the full data matrix *Y* (basis learning). Computing such an NMF decomposition based only on the data matrix *Y* is usually only a first step in a more complex processing pipeline for analyzing the data.

In this article we assume that the individual entities are labeled, e.g. ‘tumor’ or ‘non-tumor’ in a tissue typing application. In a classical two-step classification pipeline, the computation of an NMF decomposition is followed by constructing a linear or non-linear classification model (e.g. LDA or logistic regression). The label information is only used in the classification step, which is why this step is called a ‘supervised’ learning step, whereas the NMF decomposition is performed ‘unsupervised’.

In the following, we will first motivate the application of NMF methods for MSI-based tumor typing, summarize the basic known results on unsupervised NMF methods and corresponding algorithms, and outline the role of NMF in typical two-step classification schemes. The supervised NMF approach will then be described in Section 3.

### 2.1 Non-negative matrix factorization for tumor typing

**Fig. 1.**
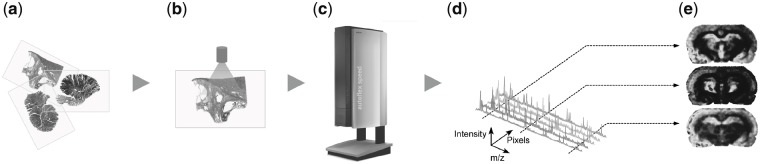
Schematic MALDI MSI workflow. Tissue sections (a) are subjected to sample preparation including deparaffination, antigen retrieval, on-tissue tryptic digestion and matrix application (b). Prepared tissue sections are inserted into the MALDI MSI instrument (c) and mass spectra (d) are acquired. When fixing single *m*/*z* values, the intensities in the measurement area can be visualized as *m*/*z* images (e), reflecting the molecular distribution of peptides with corresponding masses

A typical MALDI MSI experiment results in a data matrix *Y* consisting of measured spectra for different spatial locations of a tissue section or TMA (Fig. 1). The data matrix consists of data vectors $Y_{i,•}\in R_{\geq 0}^{m},\;i=1,\ldots n$, each one representing the spectrum measured at the spatial position with index *i* (see Supplementary Appendix C, for details).

The application of NMF methods for tumor typing is motivated by the assumption that only a comparatively small number *p* of metabolic processes or protein structures are represented in the dataset. It is hence feasible to assume that *p* spectral patterns $X_{k,•}\in R_{\geq 0}^{m},\;k=1,\ldots ,p$ with p≪min⁡(n,m) are sufficient for approximating the full dataset, and that there exist coefficients $K_{ik}\in R_{\geq 0}$ such that $Y_{i,•}\approx \sum_{k=1}^{p}K_{ik}X_{k,•}$. This results in a low-rank approximation of the data, i.e. Y≈KX. Even if not measured directly, the pseudo spectra $X_{k,•}$ (the rows of *X*) can be interpreted as mass spectra, the spatial distribution of which is given by the respective pseudo channels $K_{•,k}$ (columns of *K*). Hence, the non-negativity of *K* and *X* supports the biological interpretability, making NMF methods an ideal tool for analyzing MSI datasets and for extracting characteristic spectral patterns as a basis for classification and tumor typing.

### 2.2 Tikhonov functionals for NMF

The NMF problem, i.e. computing factors *K*, *X* such that Y≈KX, is commonly formulated as a minimization problem with a suitable discrepancy term. In this article, the discrepancy measure will always be the standard Frobenius norm, i.e. the sum of squared matrix coefficients, denoted by $||\cdot |{|}_{F}^{2}$.

To deal with the non-uniqueness of the NMF, with numerical instabilities and scaling issues, as well as to equip the matrices *K* and *X* with additional desirable properties, we incorporate additional penalty terms ${\text{ϕ}}_{\ell }$ into the cost function. Thus, the general minimization problems is of the form

(1) $$\underset{K,X\geq 0}{\min}\;\frac{1}{2}||Y-KX|{|}_{F}^{2}+\sum_{\ell }{\text{α}}_{\ell }{\text{ϕ}}_{\ell }(K,X),$$

with regularization parameters $\alpha_{\ell }\in R_{\geq 0}$ controlling the influence of the penalty terms.

The variety of potentially useful penalty terms ϕ is huge and needs to be guided by the application in mind. Here, we introduce $\ell_{2}$-regularization terms for *X* and *K* (Frobenius norm) as the simplest form of Tikhonov regularization, which avoids scaling issues and results in a better condition of the minimization problem. Moreover, an $\ell_{1}$-penalty term on *X* is introduced, increasing the sparsity of *X*, thus resulting in sparser pseudo spectra with more characteristic *m*/*z* peaks.

More precisely, this leads to the unsupervised NMF model

(2) $$\underset{K,X\geq 0}{\min}\;\frac{1}{2}||Y-KX|{|}_{F}^{2}+\text{λ}||X|{|}_{1}+\frac{\text{μ}}{2}||K|{|}_{F}^{2}+\frac{\text{ν}}{2}||X|{|}_{F}^{2}\;\text{FR}$$

with suitable regularization parameters λ,μ,ν ≥ 0, which will be abbreviated as FR (Frobenius, regularized).

Furthermore, we intend to introduce orthogonality constraints on the pseudo spectra $X_{i,•},$ such that $XX^{\text{⊺}}\approx I,$ which results in less correlated and—together with the non-negativity of *X*—sparser pseudo spectra.

However, instead of directly adding the fourth-order penalty term $||I-XX^{\text{⊺}}|{|}_{F}^{2}$ in the cost functional, we introduce a third auxiliary variable $W\in R_{\geq 0}^{p\times m}$ and split the constraint in two penalty terms. Thus, the problem is transformed to

(3) min⁡K,X,W≥0 12||Y−KX||F2+λ||X||1+μ2||K||F2+ν2||X||F2 +σ12||I−XW⊺||F2+σ22||W−X||F2, FRO

with additional regularization parameters ${\text{σ}}_{1,2}\;\geq \;0$. This NMF model is abbreviated as FRO (Frobenius, regularized, orthogonal). The special case without $\ell_{1,2}$-regularization (λ,μ,ν=0) will be referred to as FO (Frobenius, orthogonal).

### 2.3 Algorithms

All models presented in the previous section are formulated as minimization problems. These models include multiple matrix variables, i.e. *K*, *X*, as well as *W* for the FO and FRO models. The cost functionals are convex as long as only a single matrix variable is varied, but they are non-convex in the Cartesian product spaces for (*K*, *X*) and (*K*, *X*, *W*), respectively.

We follow the classical approach of majorize-minimization (MM) algorithms, (Lange, 2016) leading to alternating matrix variable updates. The key idea is to shift the minimization to surrogate functions that majorize the original cost function locally and are easier to minimize. Suitable surrogate functions for NMF-Tikhonov models as well as the resulting update rules are summarized in Supplementary Appendix A.

Rescaling the rows of *X* further improves the stability of the algorithm as well as the interpretability/comparability of the resulting spectral patterns $X_{k,•}$. Incorporating rescaling is equivalent to multiplying *X*, respectively *K*, with a diagonal matrix $D=diag\;(||X_{k,•},k=1,..,p||)$ from the left, respectively with $D^{-1}$ from the right. While rescaling is often found to be necessary with the FR model, it is less relevant with the FRO model, as the orthogonalization of *X* in the NMF decomposition implies $\ell_{2}$-normalization.

### 2.4 Classification methods based on NMF decompositions

Typical classification methods are based on two steps: In the first step, the original data vectors are transformed into some feature vectors, usually of much lower dimension. In the second step, the actual classification scheme is applied to the feature data, resulting in a binary or multi-class assignment.

Depending on the nature of the original data, different approaches for defining features can be suitable. In cases where the data vectors represent measurements of some physical quantity, correlations with suitably defined or computed basic pattern vectors are widely used. In the context of MALDI MSI data analysis, we consider features obtained by correlating a spectrum *y* (row vector of length *n*) with a set of pseudo spectra constructed by NMF methods, i.e. the row vectors of *X.* Hence, the data vector *y* is mapped to the feature vector *f* (row vector of length *p*) by $f=yX^{\text{⊺}}$.

For generating the classification model, we assume that a sufficiently large set of training data *Y* is given together with corresponding class labels *u* (ground truth), such that u(i)∈{0,1} denotes the class label of the *i*th data vector $Y_{i,•}$. The feature vectors corresponding to the full set of training data *Y* are computed by $F:=YX^{\text{⊺}}$, yielding a matrix $F\in R_{\geq 0}^{n\times p}$ of row feature vectors. Note that we only consider the case of binary classification here, although the general concepts are easily extended to the multi-class case.

In this article, we consider two different binary regression models as classification methods: LDA and logistic regression. Both models are optimized by solving a minimization problem $\min_{\text{β}}\Phi (u,F,\text{β})$, where Φ denotes some cost function measuring the discrepancy between the true class labels and the assignments of the classification model, and $\text{β}\in R^{\tilde{p}}$ denotes the set of model parameters (for details, see Supplementary Appendix B, as well as Chapter 4 in Bishop, 2006).

**Fig. 2.**
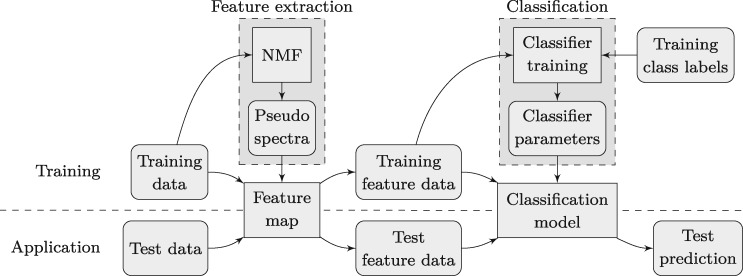
Standard process of NMF-based data classification

The overall approach of constructing and using a two-step classification method is visualized in Figure 2. For constructing the model, an annotated set of training data is used. An unsupervised NMF is computed from the training data, yielding the pseudo spectra that define the feature map. Feature vectors are then computed by applying the feature map to the training data, and are used together with the label annotations for optimizing the classification model. Once the feature map and the classification model are computed, the classification of a new data vector consists of applying the feature map to obtain a feature vector as the input for the classification model.

Combining the different unsupervised NMF decomposition schemes (FR, FO, FRO) described in this section with either LDA or logistic regression results in six different classification schemes: FR_lda, FR_log, FO_lda, FO_log, FRO_lda and FRO_log. In addition, the computation of the NMF decopompositons may or may not incorporate a rescaling of *X* and *K* in each iteration, leading to 12 different classification schemes. A characteristic subset of these methods will be evaluated in Section 4 in comparison to the supervised approaches presented in the next section.

## 3 Supervised NMF methods

### 3.1 Motivation

The NMF methods described in the previous section yield decompositions of the data matrix *Y* that provide a good approximation using only a small number *p* of basis patterns (rows of *X*) and channels (columns of *K*). Thus, we can expect *X* to consist of those spectral patterns that were found to be, in some sense, most *dominant* in the data. In the context of classification problems, however, we are not primarily interested in the most dominant components or in an accurate data approximation.

Instead, we wish to extract those spectral patterns that allow to well discriminate between spectra acquired from different tissue phenotypes, such as, for example tumor and normal tissue. The spectral features reflecting these different tissue types, however, are often subtle and much less expressed than the most dominant spectral components. Hence, they may well be considered irrelevant by a standard NMF decomposition, causing them to be suppressed in the resulting feature map $Y\mapsto YX^{\text{⊺}}$ and resulting in a decreased classification accuracy.

In the following, we will propose an extension of the standard NMF methods that allows to incorporate the a-priori label information associated with the different tissue types into the NMF minimization problem, guiding the NMF algorithm to spectral basis patterns that are most informative and relevant with respect to the classification task. The term ‘supervised NMF’ will be used to distinguish between these extended NMF methods and the standard, unsupervised methods.

### 3.2 Models and algorithms

In this section, we discuss the different supervised NMF models which are used in this article and describe shortly the respective update rules and their derivation.

The supervised NMF method differs from the standard unsupervised approach in that the classification task is done in parallel to the feature extraction. This is realized by integrating the cost function of the classification method into the NMF cost functional. Here, we consider either LDA or logistic regression as classification scheme (see Section 2.4 and Supplementary Appendix B). Using the notation of the previous sections and the cost functionals in Equations (5) and (7) in Supplementary Appendix B, leads to the basic supervised NMF models Flda

(4) $$\underset{K,X,\text{β}\geq 0}{\min}\;\frac{1}{2}||Y-KX|{|}_{F}^{2}+\frac{\text{γ}}{2}||u-YX^{\text{⊺}}\text{β}|{|}_{F}^{2}\;\text{Flda}$$

and Flog

(5) $$\underset{\begin{array}{c} K,X\geq 0,\\ \text{β} \end{array}}{\min}\;\frac{1}{2}||Y-KX|{|}_{F}^{2}+\frac{\text{γ}}{n}\left(\sum_{i=1}^{n}\log(1+{\text{e}}^{{\left[1|YX^{\text{⊺}}\right]}_{i,•}\text{β}})-u^{\text{⊺}}[1|YX^{\text{⊺}}]\text{β}\right),\;\text{Flog}$$

with the regularization parameter γ ≥ 0. Note that Flda (4) requires the non-negativity constraint on β since we assume in this model that *u* is a superposition of the correlation images $YX_{k,•}{}^{\text{⊺}}.$ However, from model Flog (5) and Equation (6) in Supplementary Appendix B.2, it is necessary for β to assume negative values in the logistic regression case in order to be able to model probabilities smaller than 0.5.

Analogously to Section 2.2, we also consider the combination of the supervised Flda NMF model with $\ell_{1}$- and $\ell_{2}$-regularization terms for *K* and *X*, as well as terms enforcing the orthogonality of *X.* These additional penalty terms have the same structure as the corresponding terms in the unsupervised case. Thus, iterative algorithms can again be determined following the MM scheme and constructing suitable surrogate functionals (see Supplementary Appendix A). This leads to the regularized supervised NMF models FRlda and FROlda with the following update rules:

K←K○YX⊺KXX⊺+μKD←K⊺KX + σ1XW⊺W + σ2X + γββ⊺XY⊺Y + νX+λX←X○K⊺Y +(σ1+σ2)W+γβu⊺YDW←W○(σ1+σ2)XW(σ1X⊺X+σ2I)β←β○XY⊺uXY⊺YX⊺β

For FRlda, let ${\text{σ}}_{1}={\text{σ}}_{2}=0$ and ignore the rule for *W.* Note that the above rules are multiplicative and preserve non-negativity as long as the matrices are initialized non-negative.

As regards model Flog (5), minimization with respect to *K* leads to the same cost functional as in Flda (4), and therefore to the same update rules for K. Due to the different structure of the logistic regression term, however, we need to follow a different approach for the updates of *X* and β. More specifically, we use a special variant of the stochastic gradient descent approach method ADADELTA (Zeiler, 2012). This adaptive method uses the information of previously computed gradients to evaluate the next step size and introduces two parameters to be chosen before performing the iterations: one parameter representing a decay rate and the second one for stability reasons. The required gradients of the cost functional in model Flog (5) with respect to *X* and β can be easily calculated analytically for this approach, which leads to ADADELTA updates ΔX and Δβ (see Zeiler, 2012 for details). To ensure the non-negativity of X, a projection step (denoted by proj(X)) is included that replaces all entries $X_{i,j}\leq 0$ with a small positive constant. This finally results in the Flog update rules:

K←K○YX⊺KXX⊺X←proj(X+ΔX)β←β+Δβ

Note that we do not add further regularization terms to the Flog functional. This has two reasons: First, adding additional regularization terms with respect to *K* could be handled by the same update rules as for Flda, whereas adding additional regularization with respect to *X* would require adapting the minimization scheme for functionals mixing logarithmic and quadratic penalty terms, which is not the focus of this article. Second, numerical tests indicate that the logistic regression term added to the Frobenius discrepancy term already yields sufficient regularization. In fact, the Flog algorithm will produce the best classification results, as seen in Section 4.

### 3.3 Combination with classification

We now assume that a supervised NMF decomposition using a variant of Flda or Flog as described in the previous section has been computed. The next step is the construction of a classifier based on these NMF vectors, i.e. a function mapping new data $\tilde{Y}\in R^{\tilde{n}\times m}$ to a class prediction $\tilde{u}\in {\left\{0,1\right\}}^{\tilde{n}}$. For that purpose we discuss two approaches.

**Integrated approach:** Directly utilizing the coefficient vector β from the supervised NMF model (Flda (4) or or Flog (5), respectively) yields the first version, which we call *integrated* classifier. It will be indicated by an _int suffix in the model names (see below). For the Flda variants one computes $\tilde{Y}X^{\text{⊺}}\text{β}\in R^{\tilde{n}}$, which assigns a real value to each new spectrum in $\tilde{Y}$. Next, entries below some threshold *t* are mapped to class 0, all other entries to class 1. The threshold *t* is determined from the training data by calculating $YX^{\text{⊺}}\text{β}\in R^{n}$ and choosing *t* such that the target performance measure (see Section 4.1) is optimized.

For the Flog variants, the regression values, computed by (8) in Supplementary Appendix B.2, can directly be interpreted as probabilities for class 1. It is hence natural to apply the threshold 0.5, i.e. entries with a probability less than 50% are predicted to belong to class 0, entries with a higher probability to class 1.

**Optimized approach:** The second approach performs the classifier training in a separate step after solving the supervised NMF model. We keep the characteristic patterns *X* determined by the supervised NMF, but we ignore the coefficient vector β. Instead we use a subsequent optimization for training an independent classifier (LDA or logistic regression) using the feature data $YX^{\text{⊺}}$ and the class labels *u.* New MALDI data $\tilde{Y}$ is then classified by applying the classifier on the feature data $\tilde{Y}X^{\text{⊺}}$. These variants will be indicated by an _lda or _log suffix.

Combining the different supervised NMF models with the above integrated or optimized classification methods leads to a large variety of classification schemes. Our main aim is to demonstrate the effect of including supervised terms in the NMF construction directly as opposed to the classical two-step approach (unsupervised NMF, classifier). To this end, we have selected the following models for comparison and evaluation: FRlda_lda, FRlda_log, FRlda_int, FROlda_lda, FROlda_log, FROlda_int, Flog_lda, Flog_log and Flog_int.

## 4 Results and discussion

In the following, we present the numerical results obtained by applying the classification schemes introduced in the previous sections to the MALDI MSI dataset described in Supplementary Appendix C. This dataset contains 4667 spectra of length 1699, it is obtained from eight TMAs of lung tumor tissues, denoted by L1 to L8. Each TMA consists of a collection of adenocarcinoma (ADC) and squamous cell carcinoma (SqCC) biopsies, proportions of both tissue types were roughly similar in all TMAs. MALDI MSI data were acquired in separate experiments for each TMA. The classification task consists in distinguishing between the tumor types ADC and SqCC, corresponding to class labels *u* = 0 and *u* = 1, respectively.

A comparison of the performance achieved on this task by the different classification schemes are given below. Moreover, the characteristics of the different NMF decompositions are investigated and compared with biological interpretations available from previous work.

### 4.1 Classification performance

In order to obtain a realistic estimation of a classification model’s performance, the available data have to be divided into a training and a test set (see Fig. 2). The results presented below were obtained by applying a standard cross-validation (CV) scheme known as *k*-fold CV. Since we wanted to evaluate the robustness of the classification methods toward technical variation between different MALDI MSI experiments, we chose to perform an 8-fold CV on TMA level.

More specifically, from the total set of TMAs L1 to L8, eight different training subsets were formed, each one consisting of seven TMAs. In each of the eight CV folds, a classification model was trained on seven training TMAs and tested on the respective remaining test TMA not included in the training set. Thus, each CV fold yields a classification result (prediction) for one of the TMAs, and the union of all predictions represents the complete prediction obtained using the respective classification scheme. Note that this CV scheme covers both the feature extraction and the classification step, as the NMF decompositions are computed on the training data only.

The accuracy of a classification result is evaluated by computing the sensitivities for both classes, i.e. the number of correctly classified spectra in each class divided by the total number of spectra in this class, and taking the average of both sensitivities. This metric, known as balanced accuracy, has the advantage of being independent of the relative proportions of the classes within the respective test data (Sokolova and Lapalme, 2009).

**Fig. 3.**
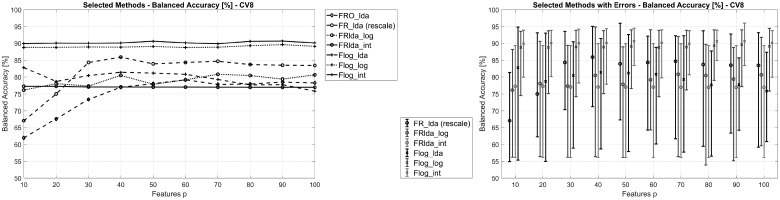
Left: Performance of selected classification schemes for different numbers of features and using 8-fold CV. Right: Performance variation for selected classification schemes for different numbers of features. Each vertical bar represents the minimum and maximum balanced accuracy achieved in the individual CV folds

In total, 13 different classification schemes were evaluated. Since the number of features *p* has a strong influence on the accuracy of many models, we varied this hyperparameter from 10 to 100 in steps of 10. The balanced accuracies for a subset of the most relevant methods is shown in Figure 3 (left), results for all methods as well as details on the regularization parameters used are presented in Supplementary Appendix D.

As can be observed, the methods Flog_int and Flog_log based on the supervised NMF model Flog (5) achieve the highest performance of approximately 90%, independent of the number of features *p.* They are followed by the FR_lda method achieving a maximum performance close to 85%. Most of the other methods achieve balanced accuracy values below 80%. Furthermore, the following detailed observations can be made (see Supplementary Appendix D):

- Among the unsupervised methods, FR is superior to the FRO model. Both achieve better results when combined with LDA classification as compared with logistic regression.
- While the supervised methods Flog_int and Flog_log outperform all others, Flog_lda performs significantly worse.
- Similarly, FR(O)lda_int and FR(O)lda_log perform better than FR(O)lda_lda, although the effect is less expressed.
- While FRlda_int and FRO_int show very similar performance, FROlda_lda and FROlda_log perform worse than their FRlda_lda/log counterparts.

In addition to the overall classification performance, we investigated the performance variation between the eight CV folds for each classification schemes. In particular where two methods show similar overall performance, it is of interest to compare the respective performance variation, as a lower variation indicates a method’s higher robustness toward biological and technical variability. As can be seen in Figure 3 (right), performance variation of Flog_int is significantly smaller than that of Flog_log, although both show almost the same stable overall performance. On the other hand, no significant differences in performance variation are noted between FRlda_int and FRlda_log.

### 4.2 Characteristic spectral patterns

The above results demonstrate that classification schemes based on the supervised NMF model Flog achieve the highest balanced accuracy values and lowest variation among all investigated methods. Most notably, these results are achieved with even a small number of features and are stable across the whole range of feature counts. This motivates the assumption that in these methods only a small number of basis vectors $X_{i,•}$ is actually relevant, and that there is only little variation in these vectors between different training sets.

**Fig. 4.**
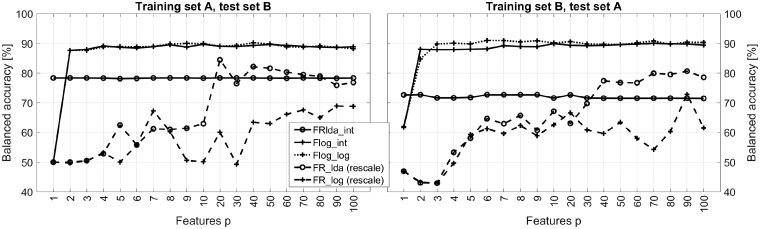
Classification performance achieved with selected supervised and unsupervised NMF models in the 2-fold CV scenario

To reduce complexity, we replace the 8-fold CV from the previous section by a 2-fold CV, in which the full dataset is split in two subsets, A={L1…L4} and B={L5…L8}. Balanced accuracy results for some of the classification schemes are shown in Figure 4. Note that in this scenario, each classifier is trained on only four out of eight TMAs, which explains the performance decrease as compared with 8-fold CV. Moreover, we extended the variation of the number of features to include p∈{1…10}.

As discussed in Section 3.2, the vector of regression weights β in Flog (5) reflects the level of influence of the spectral patterns on the logistic regression term in the NMF model. More specifically, the i + 1th entry in β is related to the *i*th pseudo spectrum $X_{i,•}$. Thus, we can interpret the values β as the relevance of the respective pseudo spectra for the classification model.

**Fig. 5.**
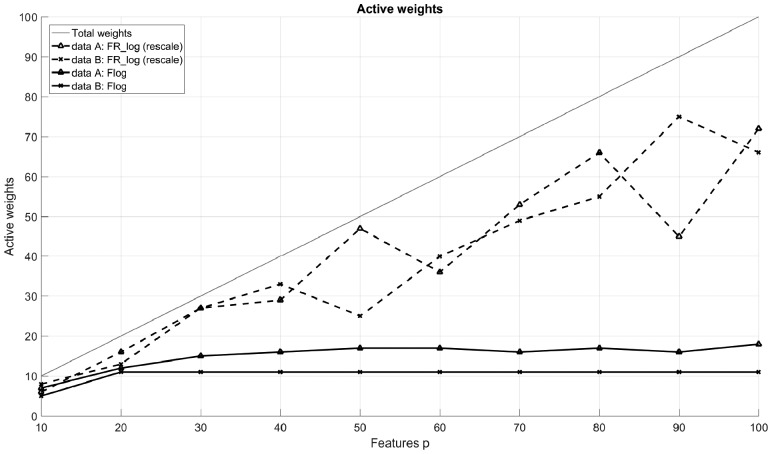
Comparison of the number of active weights for Flog and FR_log. Weights greater than 10% of the maximum absolute weight value are considered active

Figure 5 shows how the number of *active* weights, i.e. weights significantly different from zero, changes with the number of features *p.* While for Flog the number of active weights remains almost unchanged at a certain level, it continues to increase with *p* in the traditional approach FR_log and is often very close to the total number of weights. Obviously, the supervised NMF model tends to concentrate the information relevant for the classification task on a few pseudo spectra. This is not possible with the unsupervised NMF models, on the other hand, as these are independent of the subsequent classification.

A closer investigation of the pseudo spectra *X* generated by the Flog model reveals a high correlation among the basis vectors. Moreover, these patterns exhibit little dependency on the number of features and thus can be interpreted as being characteristic for the two classes ADC and SqCC.

**Fig. 6.**
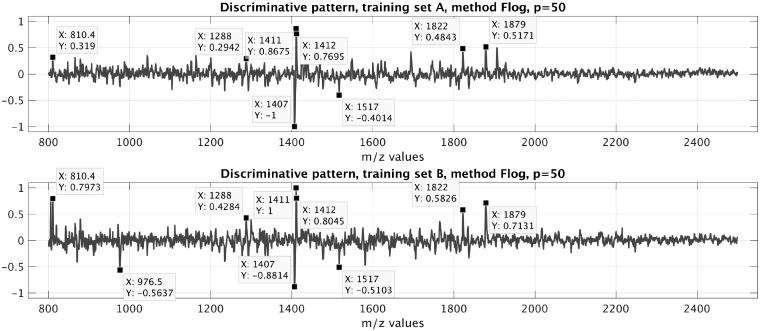
Discriminatory patterns learned by the method Flog on data A and B

To illustrate this, Figure 6 shows the weighted linear combination of pseudo spectra, given by $x_{\text{β}}=X^{\text{⊺}}\hat{\text{β}}$, which is the discriminatory pattern underlying the Flog_int classification scheme. In fact, the dominant features in this pattern align well with results published previously on the discrimination of ADC- and SqCC tissue of the lung (see Supplementary Appendix D, for further details).

## 5 Conclusion

In the past years, NMF has been established as a valuable tool for generating low-rank approximations of large datasets. Such methods have recently been applied to classification problems, where the NMF serves as a feature extraction step prior to the training of a classification model. In the context of such applications, we have presented an extension of the classical NMF framework by incorporating the class information on the training data into the NMF feature extraction step. Formally, this has been achieved in a natural way by introducing additional penalty terms into the NMF objective functional, thus being able to solve the modified problem with algorithms that are similar to those for the original problem. Moreover, this approach allows to unify the feature extraction and classifier training steps, reducing computation time and algorithmic complexity.

We have evaluated several variants of this supervised NMF approach on a challenging classification task related to MALDI MSI and its application to tumor typing in pathology. The comparison of the novel methods based on a supervised NMF decomposition with more conventional NMF-based classification schemes reveals an improved classification accuracy in some of the investigated methods. In particular, methods based on a logarithmic regression-type extension of the NMF decomposition significantly outperform all other methods in our experiments.

An in-depth analysis of the pseudo spectra and discriminative patterns generated by the supervised NMF decompositions yields a high stability of the method with respect to the training subset and the feature space dimension. Moreover, the generated patterns are amenable to a biological interpretation, thus allowing to confirm the hypothesized discriminative markers by complementary analysis techniques. In our application, we were able to extract discriminative markers that nicely match mass spectrometric markers identified by other researchers.

In future work, we plan to extend the application of this method to multi-class classification problems, as well as developing methods for making an optimal choice of the method’s hyperparameters.

## Supplementary Material

bty909_Supplementary_Appendix
