# Supplementary material for: Supervised non-negative matrix factorization methods for MALDI imaging applications
Source: Bioinformatics. 2018 Nov 5;35(11):1940–7. doi: 10.1093/bioinformatics/bty909 (PMC6546133; doi:10.1093/bioinformatics/bty909)
Supplement: bty909_Supplementary_Appendix [file bty909_supplementary_appendix.pdf]

Supervised Non-negative Matrix Factorization Methods for MALDI Imaging Applications - Supplementary Material

Johannes Leuschner Maximilian Schmidt Pascal Fernsel Delf Lachmund
Tobias Boskamp * Peter Maass

May 22, 2018— Center for Industrial Mathematics, University of Bremen, Bremen, 28359 Germany

Appendix A Algorithmic details

A.1 Surrogate functions

All models presented in this paper are formulated as minimization problems. Many models include multiple variables, e.g. the NMF models involve at least K and X, and for the supervised NMF models β is added. This constitutes a difficulty in finding optimal values, especially since the discussed cost functions in general are non-convex in the space given by the Cartesian product of the variable spaces. We approach this by separating over the variables within each minimization step of the iterative algorithms. Hereby all variables are updated alternatingly, aiming for a set of variable values minimizing the cost function.

MM algorithms (Majorize-Minimization algorithms), elaborated in Lange [2016], are a commonly used optimization strategy. The key idea is to shift the minimization to surrogate functions that majorize the original cost function locally and are desirably easier to minimize.

For a cost function f, a surrogate function $g_a(x)$ at point a is characterized by fulfilling the two conditions

$$\begin{aligned} &\text{(i)} \qquad g_a(x) \geq f(x) \quad \forall x, \\ &\text{(ii)} \qquad g_a(a) = f(a). \end{aligned}$$

By this it is easy to prove that iterating the update rule

$$x^{[k+1]} := \arg\min\, g_{x^{[k]}}(x), \qquad k = 0, 1, 2, \dots \tag{1}$$

guarantees a monotonic decrease of $f(x^{[k]})$. While this implies convergence of $f(x^{[k]})$, as stated in Lee and Seung [2001], it does not imply convergence of $x^{[k]}$, the minimizing variable itself. Also, this method possibly does not locate a global but only a local minimum. In practice, however, MM algorithms have been found to yield good solutions.

In order to use this strategy for our minimum problems, the challenge now is to find surrogate functions for the employed cost functions. There are some general approaches for obtaining surrogate functions, including applications of Jensen's inequality, the Cauchy-Schwarz inequality, and the quadratic upper bound principle. We are going to utilize the latter.

A.2 Quadratic upper bound principle

As a starting point consider the second-order Taylor polynomial of $f(x)$ around a,

$$T_{2,a}f(x) = f(a) + (x-a)^\intercal \nabla f(a) + \tfrac{1}{2}(x-a)^\intercal H_f(a)(x-a).$$

*tboskamp@uni-bremen.de

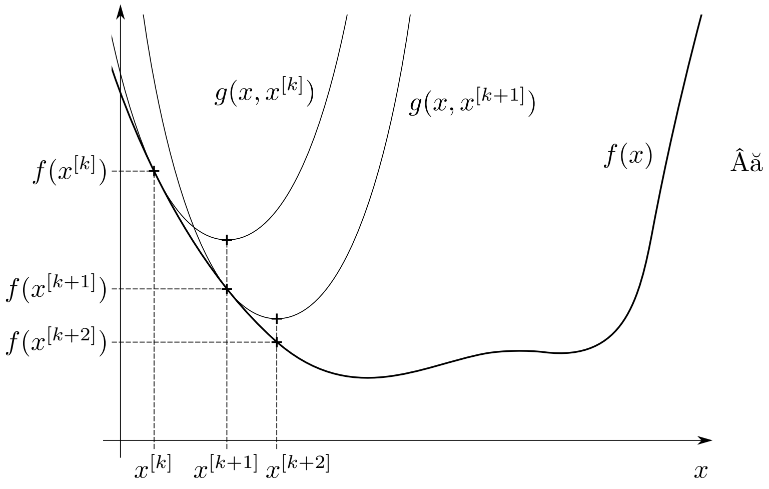

Figure A1: Two iterations of a descent achieved by the update rule (1) of the MM algorithm

Replacing the Hessian matrix $H_f(a)$ by another matrix M, whose difference to $H_f(a)$ is positive semidefinite, i.e.

$$v^\intercal (M - H_f(a))\, v \geq 0 \qquad \forall v, a,$$

generates an upper bound for $T_{2,a}f(x)$,

$$g_a(x) := f(a) + (x-a)^\intercal \nabla f(a) + \tfrac{1}{2}(x-a)^\intercal M (x-a) \;\geq\; T_{2,a}f(x). \tag{2}$$

The surrogate function condition (i) $g_a(x) \geq f(x)$ hence is fulfilled if $T_{2,a}f(x) \geq f(x)$. Since all of our cost functions are polynomials of at most second degree, the Taylor polynomials $T_{2,a}f(x)$ are equal to the functions itself. As (ii) $g_a(a) = f(a)$ obviously holds, we can use (2) as a surrogate function template.

If M is positive definite, then $g_a(x)$ is strictly convex and the calculation of its global minimum point, which is required by the update rule (1), results from solving $\nabla g_a(x) = 0$,

$$\begin{aligned} &\nabla f(a) + M(x-a) = 0 \\ \Leftrightarrow \quad & x = a - M^{-1}\nabla f(a). \end{aligned} \tag{3}$$

Note that the inverse M^{-1} is clear to exist by the positive definiteness of M.

When choosing M, it is reasonable to require M^{-1} to be straightforward to compute. Another quite important aim originates from the non-negativity constraint on x in (3) imposed when updating K or X. This can be easily satisfied if the update rule (3) is multiplicative, in the sense that it can be rewritten by only addition, multiplication and division of the used variables, and particularly not by subtraction. If this holds for all alternating update rules, the non-negativity of all variables is automatically maintained throughout the whole algorithm after choosing non-negative initial values. This avoids the need for projections into the valid spaces, which are commonly used in other optimization algorithms.

For our applications the choice of the diagonal matrix

$$M = \left[\delta_{i,j} \frac{(H_f(a)a)_i + \lambda}{a_i}\right]_{i,j}, \tag{4}$$

whose inversion is naturally trivial, will lead to multiplicative update rules. This is owing to the special relation between the gradients $\nabla f(a)$ and the Hessians $H_f(a)$. Furthermore, in our cases H_f has non-negative components, a property that ensures positive semidefiniteness of $M - H_f$ for the proposed choice of M (Blondel et al. [2008]).

In (4) the parameter λ covers the case of ℓ_1-regularization on the updated variable. Nevertheless for M to exist and to be positive definite, we need both the numerator and the denominator in (4) to be non-zero, which leads us to require not only non-negativity but rather strictly positiveness for K and X. This is not sensible from the perspective of NMF or MALDI imaging, especially since we prefer sparse pseudo spectra, but is expedient to assume during the algorithm. We technically ensure the positiveness by adding a small value to each variable component after its regular update. When finally interpreting the results, a small threshold can be used to gain actual sparsity.

A.3 Update rules

To obtain the update rules for the required variables, firstly the Hessian is calculated and substituted in (4). This is then substituted in (3), which is rearranged conveniently. The resulting update rules for models *F, FRO, FROlda*, and *Flog* are listed below.

F

$$\begin{aligned} K &\leftarrow K \circ \frac{YX^\intercal}{KXX^\intercal} \\ X &\leftarrow X \circ \frac{K^\intercal Y}{K^\intercal KX} \end{aligned}$$

FRO

$$\begin{aligned} K &\leftarrow K \circ \frac{YX^\intercal}{KXX^\intercal + \mu K} \\ X &\leftarrow X \circ \frac{K^\intercal Y + (\sigma_1 + \sigma_2)W}{K^\intercal KX + \sigma_1 XW^\intercal W + \sigma_2 X + \nu X + \lambda} \\ W &\leftarrow W \circ \frac{(\sigma_1 + \sigma_2)X}{W(\sigma_1 X^\intercal X + \sigma_2 I)} \end{aligned}$$

FROlda

$$\begin{aligned} K &\leftarrow K \circ \frac{YX^\intercal}{KXX^\intercal + \mu K} \\ X &\leftarrow X \circ \frac{K^\intercal Y + (\sigma_1 + \sigma_2)W + \gamma\beta u^\intercal Y}{K^\intercal KX + \sigma_1 XW^\intercal W + (\sigma_2 + \nu)X + \gamma\beta\beta^\intercal XY^\intercal Y + \lambda} \\ W &\leftarrow W \circ \frac{(\sigma_1 + \sigma_2)X}{W(\sigma_1 X^\intercal X + \sigma_2 I)} \\ \beta &\leftarrow \beta \circ \frac{XY^\intercal u}{XY^\intercal YX^\intercal \beta} \end{aligned}$$

Flog

$$\begin{aligned} K &\leftarrow K \circ \frac{YX^\intercal}{KXX^\intercal} \\ X &\leftarrow \operatorname{proj}(X + \Delta X) && \text{(Gradient based update, projection on } \mathbb{R}_+\text{)} \\ \beta &\leftarrow \beta + \Delta\beta && \text{(Gradient based update)} \end{aligned}$$

Appendix B Linear and logistic regression for binary classification

B.1 Linear discriminant analysis

A standard procedure for generating a classification model based on an NMF decomposition, as mentioned in Section 2.4 (main article), is to approximate given binary class labels $u \in \{0,1\}^n$ by a linear combination of the correlations $YX_{k,\bullet}{}^\intercal$ and leads to the matrix $F := YX^\intercal \in \mathbb{R}_{\geq 0}^{n\times p}$, which contains row-wise the features vectors $Y_{i,\bullet}X^\intercal$ of length p. Thus, coefficients $\beta \in \mathbb{R}^p$ have to be determined, such that $u \approx \sum_{k=1}^p \beta_k F_{\bullet,k} = YX^\intercal\beta$. Due to the optimization method and the multiplicative update rules, these coefficients are automatically non-negative in the case of the LDA, so that u is a superposition of the correlations $F_{\bullet,k}$. To also allow the modeling of class labels equal to zero, we therefore omit a strict positive bias and consider the unbiased case in contrast to default LDA regression models.

The estimation of β is typically done with a least squares method and leads to the minimization problem

$$\min_\beta \|u - YX^\intercal\beta\|_F^2. \tag{5}$$

The minimization using the available annotated training data yields a suitable parameter set $\hat{\beta}$ and a corresponding characteristic vector $\hat{x} = X^\intercal\hat{\beta}$. The actual classification of a new data set y is now straightforward, it just requires to compute the scalar product $c = y\hat{x}$ and the resulting classification is obtained by a binary threshold on c.

One great advantage of the linear regression model is its simplicity and the manageability. However, the fundamental drawback of this method is the fact that u is a binary coded target variable which is in contrast to the input data F, whose entries lie typically in $\mathbb{R}_{\geq 0}$. The linear regression model is commonly not the natural choice to approximate binary output data with a continuous input. We therefore consider logistic regression as an alternative approach.

B.2 Logistic regression

The logistic regression method represents a more natural approach to model binary output variables. It is a special case of the general logistic regression for two classes and is also known as the *logit* model. Here, we use the standard biased case of the logit approach and define accordingly $F := [1|YX^\intercal]$. The basic idea is to model the posterior probabilities $\pi_i = \mathrm{P}(u_i = 1)$ for the occurrence of $u_i = 1$ by applying the standard logistic function $h(x) = (1 + e^{-x})^{-1} \in [0, 1]$ to the so called linear predictors $F_{i,\bullet}\beta$, yielding the regression model

$$\pi_i = h(F_{i,\bullet}\beta) = \frac{1}{1 + \exp(-F_{i,\bullet}\beta)}. \tag{6}$$

As mentioned in Section 3.2 (main article), we will also allow negative entries in β to be able to model probabilities lower than 0.5.

The estimation of the parameter β is typically done by applying the maximum likelihood method. Since the output variable u_i underlies the Bernoulli distribution, it follows for the likelihood function $L(\beta) = \prod_{i=1}^n \pi_i^{u_i}(1-\pi_i)^{1-u_i}$. This leads finally to the minimization of the negative log-likelihood l^- to get an estimator for the parameter β, such that

$$\min_\beta l^-(\beta) = \min_\beta \sum_{i=1}^n \log(1 + \exp(F_{i,\bullet}\beta)) - u^\intercal F\beta. \tag{7}$$

The prediction on new datasets $\tilde{Y}$ is done by using (6) and calculating

$$\tilde{\pi}_i = \frac{1}{1 + \exp(-[1|\tilde{Y}X^\intercal]_{i,\bullet}\beta)}. \tag{8}$$

The main advantage over the linear regression method is that the values of the prediction can be directly interpreted as probabilities for the occurrence of tumorous regions in the tissue sample.

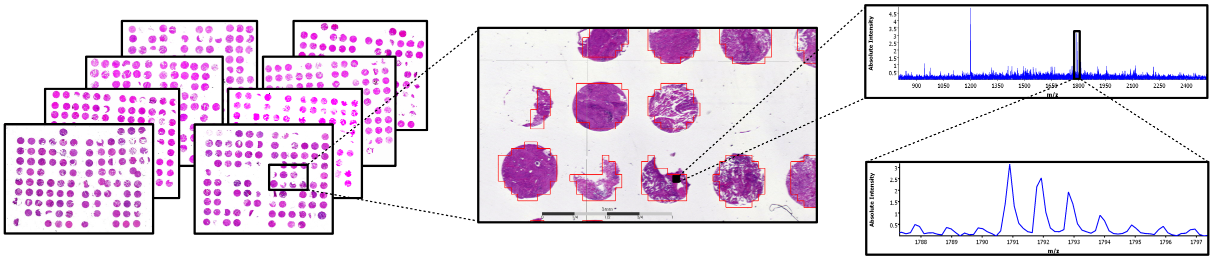

Figure C2: Schematic data structure. HE images of 8 TMAs (left), each containing multiple core biopsies of ADC or SqCC patients (middle). For each core several MALDI TOF spectra are collected (upper right). The close-up shows an isotopic pattern for a peptide at m/z 1790.9 (lower right).

Appendix C MALDI imaging and test data

Mass spectrometry imaging (MSI), in particular matrix-assisted laser desorption/ ionization (MALDI) MSI, is a label-free technique for spatially resolved molecular analysis of biological tissue samples with a broad range of applications in pharmaceutical and biomedical research. With recent technological advances in acquisition speed and robustness, applications of MALDI MSI in pathological diagnostics became feasible, where this method can help to, for example, characterize tumor tissue, delineate tumor regions or identify tumor subtypes (Groseclose et al. [2008], Seeley and Caprioli [2011]).

In MALDI MSI, a pulsed laser beam is focused at a series of spots (pixels) covering a tissue sample with a spatial resolution ranging from 5 to 250 µm. Biomolecules over a wide mass range are desorbed from the tissue surface, ionized and fed into a mass spectrometer. The resulting dataset consists of a complete mass spectrum with up to 1 million spectral intensity values for each tissue spot, yielding extremely high-dimensional multispectral images for arbitrary ion masses.

The determination of spatial tissue regions with different biological characteristics requires the extraction of biologically meaningful information out of the spectral data, i.e. the identification of “spectral fingerprints” associated with different tissue phenotypes. A successful extraction of such biological information heavily depends on a careful sample preparation and data acquisition, including sample collection and fixation, several preparation steps, as well as matrix deposition and the actual MALDI measurement. For details on this process we refer to Oetjen et al. [2016] and references therein.

In a common workflow, formalin-fixed paraffin-embedded (FFPE) tissue is subjected to a tryptic digestion step, resulting in the decomposition of proteins into smaller fragments (peptides). As a consequence, the spectral fingerprint of a protein biomarker typically consists of a characteristic pattern of spectral peaks corresponding to different peptides, as well as molecular modifications and isotopes. This observation, together with the fact that spectral intensities are strictly non-negative, motivates the use of non-negative matrix factorization (NMF) algorithms for analyzing MALDI MSI datasets.

In the following we evaluate the proposed methods on a collection of FFPE lung tumor tissue samples, including biopsies of adenocarcinoma (ADC) and squamous cell carcinoma (SqCC) (Kriegsmann et al. [2016]). Differentiation between these two frequent subtypes of non-small cell lung cancer is of high relevance for therapy selection and patient management. Diagnostic accuracy of current procedures based on immunohistochemical (IHC) stainings, however, is limited, resulting in a demand for novel analytical tumor typing methods. The applicability of MALDI MSI to this task has been demonstrated in Kriegsmann et al. [2016], where the presence of five different marker peptides belonging to four proteins has been validated.

Tissue samples for our study were provided by the tissue bank of the National Center for Tumor Diseases (NCT, Heidelberg, Germany) in accordance with the regulations of the local ethics committee. Cylindrical tissue cores of 168 ADC and 136 SqCC were assembled to 8 tissue microarray (TMA) blocks. Tissue sections were cut from all TMA blocks and processed according to a typical tissue preparation protocol established for tryptic peptide imaging (Casadonte and Caprioli [2011]).

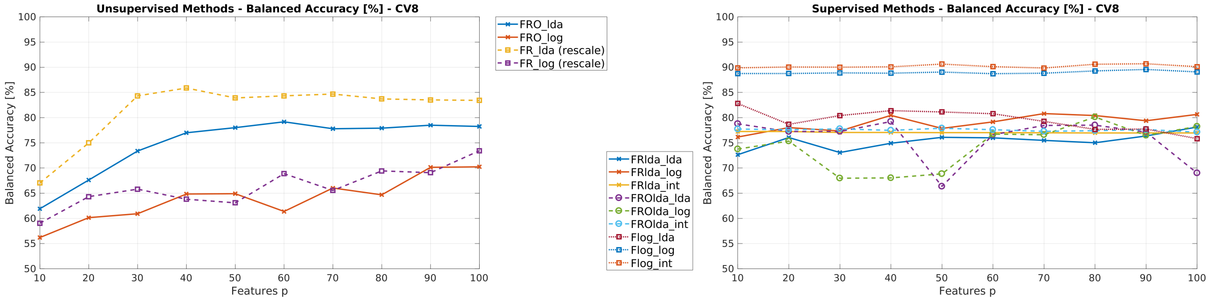

Figure D3: Performance of classification schemes based on unsupervised (left) and supervised (right) NMF models, with number of features ranging from 10 to 100.

Model	λ	μ	ν	$\sigma_{1/2}$	γ
FRO	1.0×10^{-4}	1.0×10^{-6}	1.0×10^{-9}	0.1	–
FR (rescale)	0	0	0	–	–
FRlda	1.0×10^{-5}	1.0×10^{-4}	1.0×10^{-7}	–	1
FROlda	1.0×10^{-3}	1.0×10^{-4}	1.0×10^{-7}	0.3	1
Flog	0	0	0	–	1

Table D1: Regularization parameters for the NMF models

MALDI imaging data was acquired on an Autoflex speed MALDI-TOF/TOF mass spectrometer (Bruker Daltonik) in positive ion reflector mode. Spectra were measured in the mass range 500–5000 m/z at 150 µm spatial resolution (Figure C2). After MALDI MSI data acquisition, the sections were washed and hematoxilyn-eosin (HE) stained. Tumor status and typing for all cores were confirmed by standard histopathological examination and detailed annotations were created indicating subregions containing tumor cells.

Spectral data of all TMAs was loaded into SCiLS Lab (version 2016b, Bruker Daltonik) and the software's default baseline correction as well as total ion count (TIC) normalization was applied. For subsequent processing, the data was exported to MATLAB (version 2016b, Mathworks) and spectra outside of the annotated tumor subregions were discarded. The remaining 4667 spectra were cropped to the mass range 800–2500 m/z and resampled to intervals of 0.4 Da width centered around expected peptide masses according to the averagine model (Senko et al. [1995]). Thus, the dimensionality of the data was reduced to 1699 intensity values per spectrum. In the following, the complete spectral dataset is represented by a matrix Y, where spectra are stored as rows and columns correspond to m/z images. Such MSI data sets are a special case of hyperspectral imaging data, where the channels of the hyperspectral data are the m/z images of the MSI data.

Appendix D Detail results and correlation analysis

Detailed performance results for all investigated classification schemes are shown in Figure D3. The regularization parameters used for the different NMF models are listed in Table D1.

Classification schemes based on the supervised NMF methods (in particular with the *Flog* model) require a surprisingly low number of features for reaching convergence in terms of classification accuracy. Indeed, a closer investigation of the pseudo spectra X generated by the *Flog* model reveals a high correlation among the basis vectors, as demonstrated in Figure D4 for the case of $p = 20$ features. In fact, as much as 15 rows of X essentially represent two distinct patterns, clearly associated with positive and negative signs of β. Moreover, these patterns exhibit little dependency on the number of features (Figure D5), and thus can be interpreted as being characteristic for the two tissue classes ADC ($\beta < 0$) and SqCC ($\beta > 0$).

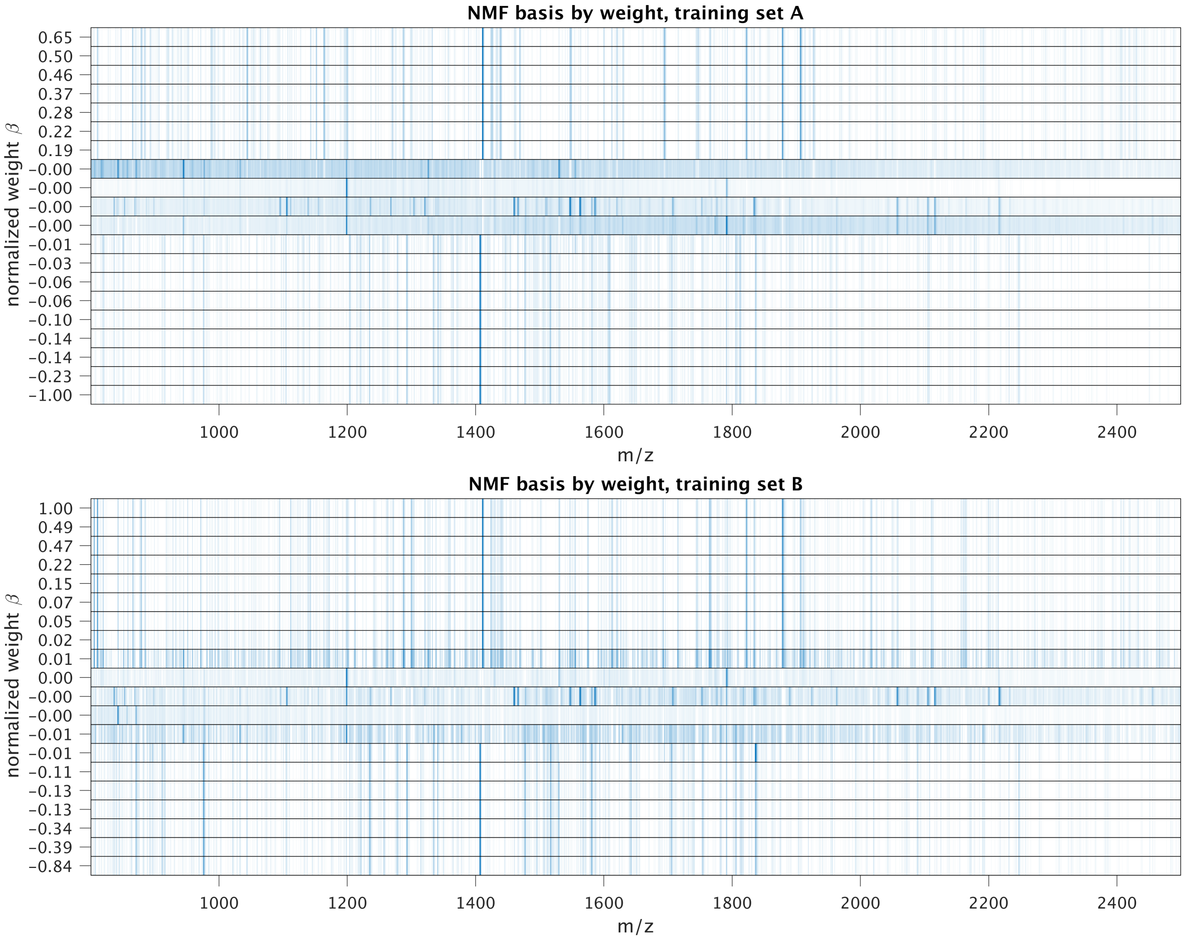

Figure D4: Gel view of pseudo spectra X generated by the *Flog* NMF decomposition of training data A and B at $p = 20$ features. Rows are sorted by corresponding regression weights β.

For comparison of the observed characteristic patterns with results published previously on the discrimination of adeno- and squamous cell carcinoma tissue of the lung, we investigate prominent features in the weighted linear combination of pseudo spectra, given by $x_\beta = X^\intercal \hat{\beta}$. The resulting discriminatory patterns are shown in Figures 6 (main article) and D6 for the supervised NMF models *FRlog* and *Flda*, resp. Note that for the *Flda* model, we can let $\hat{\beta} = \beta$, whereas in the *Flog* model, the vector β includes one leading component representing a constant offset (see equation Flog (5) in the main article.) Thus, for *Flog*, $\hat{\beta}$ equals β with the first component removed.

The model *FRlda* includes an ℓ_1-penalty term on X that induces sparsity in the pseudo spectra. As a result, the discriminatory patterns for this model feature only very few peaks, and only the peaks at $m/z = 1411$ and 1412 occur in both patterns learned on data A and B, resp. By comparison with results published in Kriegsmann et al. [2016], we attribute these to the monoisotopic peak of a peptide of the CK5 protein ($m/z = 1410.7$) and it's second isotopic peak. Moreover, the peaks at $m/z = 1879$ and 1907 that occur in only one of the two patterns can most likely be attributed to peptides of the proteins CK15 (monoisotopic $m/z = 1877.9$) and HSP27 (monoisotopic $m/z = 1905.9$). In fact, all three proteins have been shown to be indicative for squamous cell carcinoma as opposed to adenocarcinoma in the lung, matching well the strong expression in the above discriminatory patterns.

The discriminatory patterns computed based on the *Flog* model, on the other hand, show a different

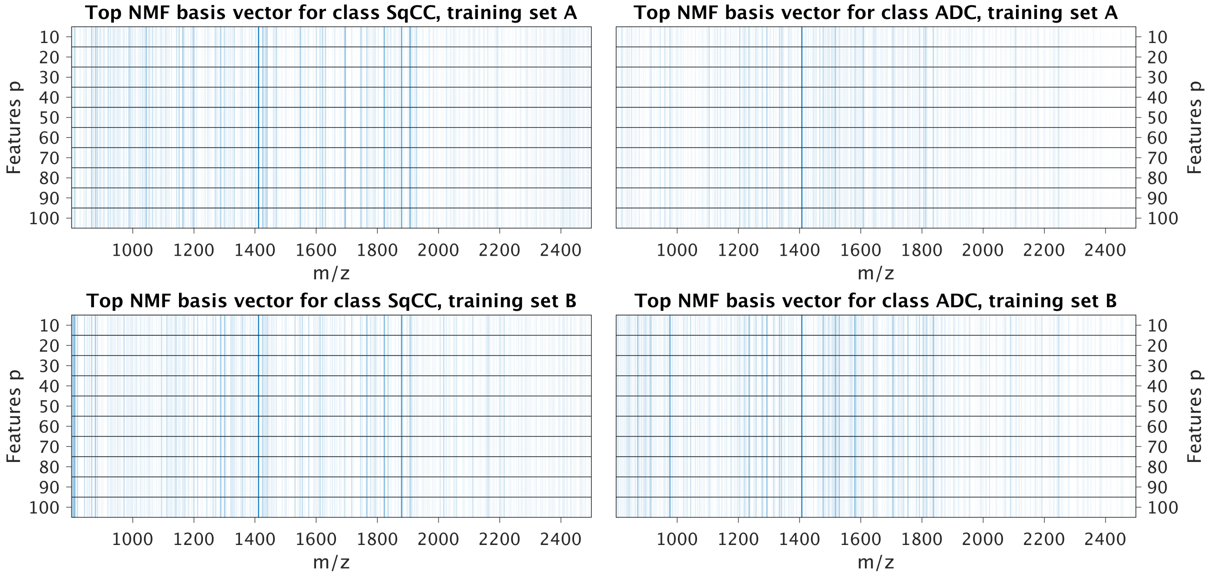

Figure D5: Comparison of the two opposite pseudo spectra X associated with the largest (positive, indicative for SqCC) and smallest (negative, ADC) weights β for different numbers of features, generated by the *Flog* NMF decomposition of training data A and B.

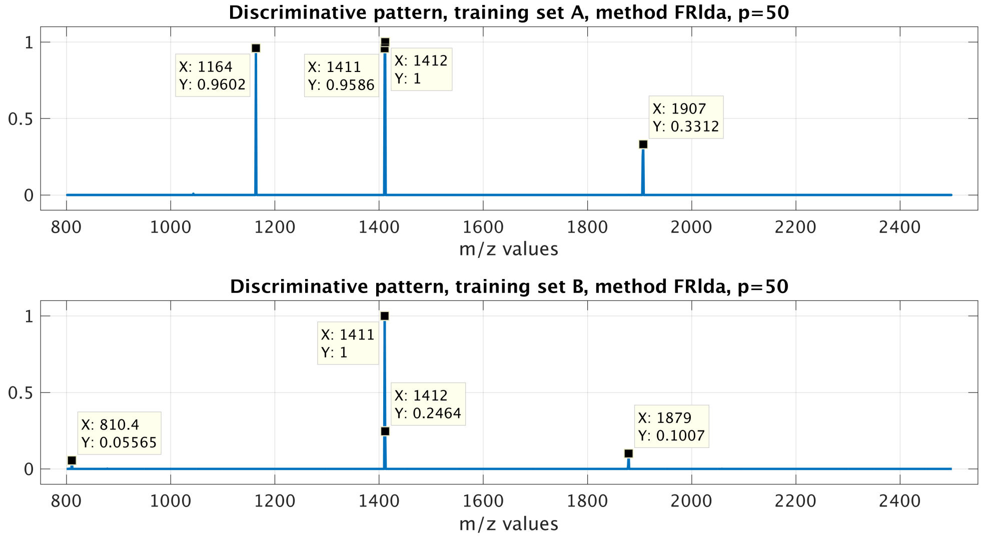

Figure D6: Discriminatory patterns learned by the method *FRlda* on data A and B.

characteristic and contain a significant background noise, which is most likely due to the missing sparsity regularization term in this method. Moreover, since in the *Flog* model β is allowed to be negative, both positive (indicative for SqCC) and negative (indicative for ADC) peaks are seen. Only little difference can be observed between the discriminatory patterns learned on data A and B. In addition to peaks at $m/z = 1411/1412$ as well as $m/z = 810.4$ and 1879 also occurring in the *FRlda* patterns, peaks at $m/z = 1407$ and 1822 can be observed. The former, having a negative direction, is most likely related to a peptide of the CK7 protein (monoisotopic $m/z = 1406.7$), which was demonstrated to be indicative for adenocarcinoma in the lung (Kriegsmann et al. [2016]). The other peak can be attributed to a different

peptide of the above mentioned CK15 protein (monoisotopic $m/z = 1821.9$) indicative for squamous cell carcinoma.

References

V. D. Blondel, N.-D. Ho, P. Dooren, et al. Weighted nonnegative matrix factorization and face feature extraction. In *Image and Vision Computing*. Citeseer, 2008.

R. Casadonte and R. M. Caprioli. Proteomic analysis of formalin-fixed paraffin embedded tissue by MALDI imaging mass spectrometry. *Nature protocols*, 6(11):1695, 2011.

M. R. Groseclose, P. P. Massion, P. Chaurand, and R. M. Caprioli. High-throughput proteomic analysis of formalin-fixed paraffin-embedded tissue microarrays using MALDI imaging mass spectrometry. *Proteomics*, 8(18):3715–3724, 2008.

M. Kriegsmann, R. Casadonte, J. Kriegsmann, H. Dienemann, P. Schirmacher, J. H. Kobarg, K. Schwamborn, A. Stenzinger, A. Warth, and W. Weichert. Reliable entity subtyping in non-small cell lung cancer by matrix-assisted laser desorption/ionization imaging mass spectrometry on formalin-fixed paraffin-embedded tissue specimens. *Molecular & Cellular Proteomics*, 15(10):3081–3089, 2016.

K. Lange. *MM Optimization Algorithms.* Other Titles in Applied Mathematics. Society for Industrial and Applied Mathematics, 2016.

D. D. Lee and H. S. Seung. Algorithms for non-negative matrix factorization. In T. K. Leen, T. G. Dietterich, and V. Tresp, editors, *Advances in Neural Information Processing Systems 13*, pages 556–562. MIT Press, 2001.

J. Oetjen, D. Lachmund, A. Palmer, T. Alexandrov, M. Becker, T. Boskamp, and P. Maass. An approach to optimize sample preparation for MALDI imaging MS of FFPE sections using fractional factorial design of experiments. *Analytical and Bioanalytical Chemistry*, 408(24):6729–6740, 2016.

E. H. Seeley and R. M. Caprioli. MALDI imaging mass spectrometry of human tissue: method challenges and clinical perspectives. *Trends in Biotechnology*, 29(3):136–143, 2011.

M. W. Senko, S. C. Beu, and F. W. McLafferty. Determination of monoisotopic masses and ion populations for large biomolecules from resolved isotopic distributions. *J Am Soc Mass Spectrom*, 6:229–233, 1995.
